# *Mycoplasma hyopneumoniae* resides intracellularly within porcine epithelial cells

**DOI:** 10.1038/s41598-018-36054-3

**Published:** 2018-12-06

**Authors:** B. B. A. Raymond, L. Turnbull, C. Jenkins, R. Madhkoor, I. Schleicher, C. C. Uphoff, C. B. Whitchurch, M. Rohde, S. P. Djordjevic

**Affiliations:** 1 The ithree institute, University of Technology Sydney, Ultimo, NSW 2007 Australia; 2 NSW Department of Primary Industries, Elizabeth Macarthur Agricultural Institute, PMB 8, Camden, NSW Australia; 3 Microscopy, Helmholtz Centre For Infection Research, Braunschweig, Germany; 4 Leibniz-Institute DSMZ-German Collection of Microorganisms and Cell Cultures, Braunschweig, Germany

## Abstract

Enzootic pneumonia incurs major economic losses to pork production globally. The primary pathogen and causative agent, *Mycoplasma hyopneumoniae*, colonises ciliated epithelium and disrupts mucociliary function predisposing the upper respiratory tract to secondary pathogens. Alleviation of disease is reliant on antibiotics, vaccination, and sound animal husbandry, but none are effective at eliminating *M. hyopneumoniae* from large production systems. Sustainable pork production systems strive to lower reliance on antibiotics but lack of a detailed understanding of the pathobiology of *M. hyopneumoniae* has curtailed efforts to develop effective mitigation strategies. *M. hyopneumoniae* is considered an extracellular pathogen. Here we show that *M. hyopneumoniae* associates with integrin β1 on the surface of epithelial cells via interactions with surface-bound fibronectin and initiates signalling events that stimulate pathogen uptake into clathrin-coated vesicles (CCVs) and caveosomes. These early events allow *M. hyopneumoniae* to exploit an intracellular lifestyle by commandeering the endosomal pathway. Specifically, we show: (i) using a modified gentamicin protection assay that approximately 8% of *M. hyopneumoniae* cells reside intracellularly; (ii) integrin β1 expression specifically co-localises with the deposition of fibronectin precisely where *M. hyopneumoniae* cells assemble extracellularly; (iii) anti-integrin β1 antibodies block entry of *M. hyopneumoniae* into porcine cells; and (iv) *M. hyopneumoniae* survives phagolysosomal fusion, and resides within recycling endosomes that are trafficked to the cell membrane. Our data creates a paradigm shift by challenging the long-held view that *M. hyopneumoniae* is a strict extracellular pathogen and calls for *in vivo* studies to determine if *M. hyopneumoniae* can traffic to extrapulmonary sites in commercially-reared pigs.

## Introduction

*Mycoplasma hyopneumoniae* is the etiological agent of enzootic pneumonia and a primary pathogen in the porcine respiratory disease complex^1^. Globally, porcine enzootic pneumonia is widespread and inflicts significant economic losses to pork production. Losses are incurred via reduced growth rate and feed conversion efficiency, costs for treatment and vaccination, and excessive morbidity and mortalities resulting from the combined effects of multiple respiratory pathogens. *M. hyopneumoniae* influences the ciliary beat frequency, induces ciliostasis and causes epithelial cell death, culminating in a devastating assault on the mucociliary escalator and an excessive host immune response in the lungs^2–5^. *M. hyopneumoniae* colonises cilia that project into the luminal surface of epithelial cells of the respiratory tract and is rarely found associated with the epithelial cell body^5,6^. These observations suggest that *M. hyopneumoniae* recognises receptors expressed on the surface of cilia but are limited in their presentation on the cell body. *M. hyopneumoniae* attaches to cilia via highly expressed, structurally and functionally complex^7^ adhesins that are present on the cell surface of *M. hyopneumoniae* as a diverse combination of cleavage fragments that bind multiple host molecules including highly sulphated glycosaminoglycans, fibronectin and plasminogen^8–21^.

Strategies that are implemented to control infection by *M. hyopneumoniae* include vaccination (predominantly with bacterin formulations); antibiotic therapy and herd management (high standards in hygiene, all-in-all-out production models and swiss de-population with re-stocking from herds considered free of *M. hyopneumoniae*). Combinations of these strategies are effective, however, there remains a pressing need to lower reliance on antibiotics to control infection in intensively reared animal production systems^22–25^. Sound herd management is challenged by the need to identify pig production operations free of *M. hyopneumoniae* and it remains a challenge to identify subclinically-infected and carrier animals. Ultimately, further investigation into the survival mechanisms of this important porcine pathogen is required to aid in the development of future strategies to prevent and control transmission.

It is well known that numerous mycoplasma species can invade host cells^26–29^, and although it has historically been characterised as a strict extracellular pathogen, *M. hyopneumoniae* has been cultured from the liver, spleen, kidneys and bronchial lymph nodes of pigs infected experimentally with *M. hyopneumoniae*^30–32^. However, it is not known if *M. hyopneumoniae* colonises tissue sites distal to the respiratory tract in commercially-reared herds. Interestingly, *M. hyopneumoniae* has been isolated in pure culture from both pericardial and synovial joint fluids in slaughter-age commercial pigs with fibrinous pericarditis^33^. It is not known how *M. hyopneumoniae* traffics to these sites nor is it known if *M. hyopneumoniae* can invade epithelial cells and trigger cellular uptake pathways.

In this study for the first time, we show that *M. hyopneumoniae* cells interact with integrin β1 via fibronectin and colocalise in a manner that promotes cellular uptake via caveosomes and clathrin-coated vesicles. We monitored the cellular events that depict *M. hyopneumoniae* trafficking via the endocytic pathway, and escaping phagolysosomal fusion, before residing free in the cytoplasm. Collectively, our data have significant implications for detecting animals infected with *M. hyopneumoniae* and for development of therapies to eliminate this difficult-to-control pathogen.

## Results

### *M. hyopneumoniae* resides intracellularly within epithelial cells

**Figure 1:**
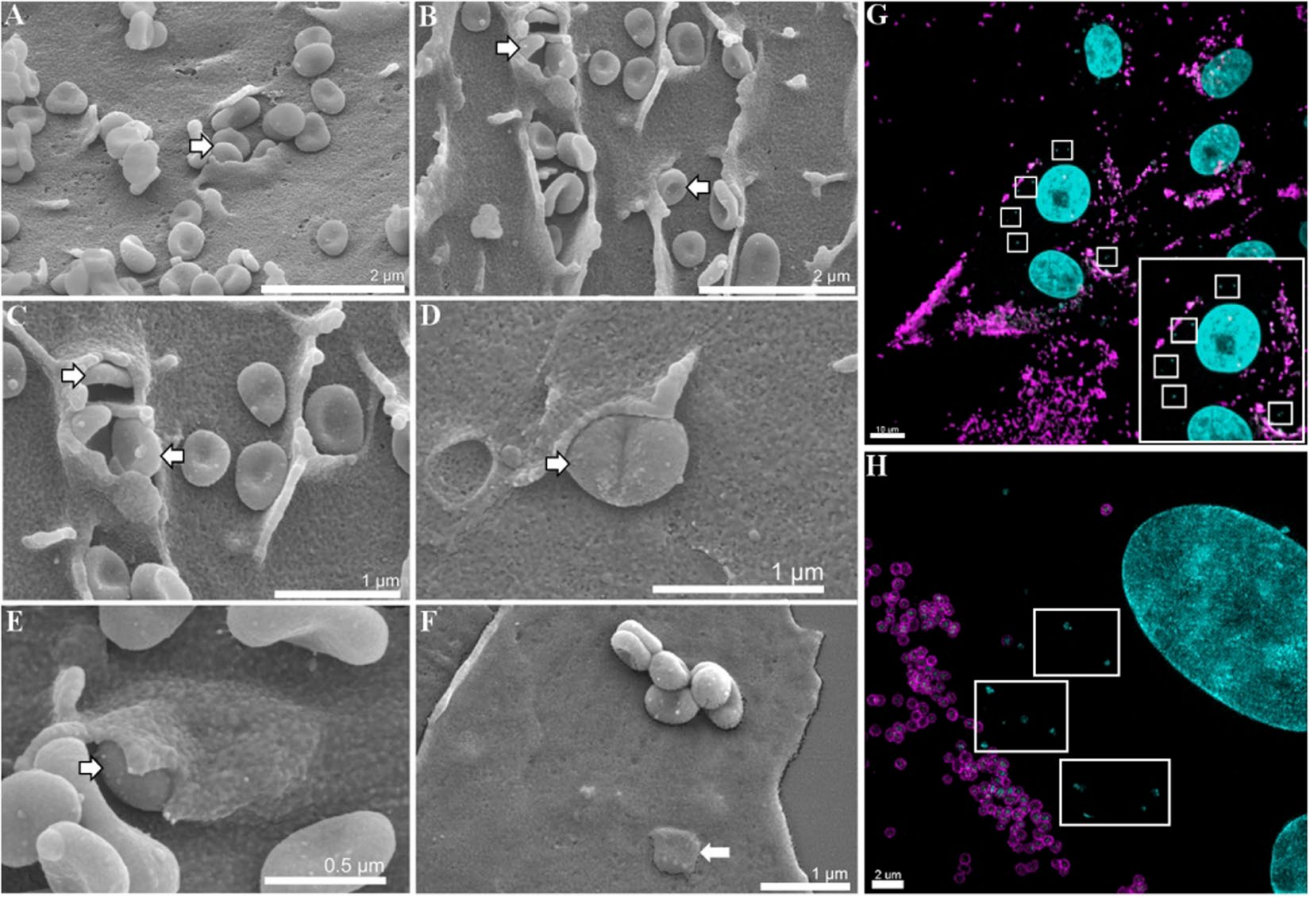
*M. hyopneumoniae* resides intracellularly within PK-15 cells. (**A**–**F**) SEM of *M. hyopneumoniae* cells interacting with the surface and becoming internalised within PK-15 monolayers (white arrows with black outlines). (**A**–**E**) *M. hyopneumoniae* cells being engulfed by PK-15 cells. Microvilli/filopodia form cell surface projections that envelope the *M. hyopneumoniae* cells. (**F**) A small cluster of *M. hyopneumoniae* cells adhering to the monolayer and one cell (white arrow) which has become engulfed. Scale bars in A-F are 2 µm, 2 µm, 1 µm, 1 µm, 500 nm, and 1 µm respectively. (**G**,**H**) Immunofluorescence images of PK-15 cells infected with *M. hyopneumoniae*. Extracellular *M. hyopneumoniae* cells were labelled with F2_P94-J_ antisera conjugated to CF 488 (magenta). Nucleic acids were stained with DAPI (cyan). Intracellular *M. hyopneumoniae* cells imaged using confocal microscopy (**G**) and 3D-SIM (**H**) are outlined by white boxes. Inset (bottom right corner) in panel G depicts a region showing intracellular bacteria (outlined in white).

In order to gather insight into how *M. hyopneumoniae* colonises host epithelial cell surfaces, scanning electron microscopy (SEM) was used to visualise the pattern of adherence to porcine kidney epithelial cells (PK-15) after 16 h. PK-15 cells have been used extensively as a model system for studying host-*M. hyopneumoniae* interactions^14,34–36^. SEM images showed both small clusters and individual *M. hyopneumoniae* cells associated intimately with the cell surface of PK-15 cell monolayers (Fig. 1A–D). These adhering bacterial cells are encapsulated by cell surface projections via a process that resembles macropinocytosis (Fig. 1A–E), which occasionally leads to the complete engulfment of the bacteria (Fig. 1F). Using immunofluorescence microscopy, we were able to confirm that these engulfed bacteria were indeed *M. hyopneumoniae* cells using anti-F2_P94-J_ antiserum, which is specific for *M. hyopneumoniae*. We double-labelled *M. hyopneumoniae*-infected PK-15 monolayers using F2_P94-J_ antisera conjugated to either CF 488 or CF 568 before and after permeabilisation, respectively. From this, extracellularly adhering bacteria were double-labelled (appearing yellow/green, Supplementary Fig. S1), while those cells residing intracellularly were singularly labelled (appearing red, Supplementary Fig. S1). Once we confirmed that these intracellular bacteria were *M. hyopneumoniae*, we replaced the secondary labelling step with the membrane impermeable dye DAPI, as it reliably stains *M. hyopneumoniae* nucleic acids^35^ while also reducing background staining. Extracellularly adhering *M. hyopneumoniae* cells (labelled with anti-F2_P94-J_ antisera; magenta in Fig. 1G,H) were readily distinguishable from those residing intracellularly (stained with DAPI; cyan in Fig. 1G,H). Confocal laser scanning microscopy (CLSM) and 3D-Structured Illumination Microscopy (3D-SIM) images of these samples depict extracellular, F2_P94-J_-labelled *M. hyopneumoniae* cells adhering to PK-15 cells, and numerous intracellular bacteria stained solely with DAPI (Fig. 1G,H). In uninfected control monolayers that were stained with DAPI post-permeabilisation, only the nuclei of the PK-15 cells were visible (Supplementary Fig. S2). This confirmed that the staining technique did not stain nucleic acids in the cytoplasm of PK-15 monolayers and could be used to distinguish between extracellular and intracellular bacteria. To determine the number of intracellular *M. hyopneumoniae* cells, we applied a modified and optimised version of the gentamicin protection assay^37^. This showed that approximately 1 in every 12 (8%) *M. hyopneumoniae* cells that adhered to PK-15 cells reside intracellularly and are potentially invasive (data not shown). Although it is important to note that the gentamicin protection assay is a crude quantitative measure of cell invasion, we have routinely observed *M. hyopneumoniae* cells residing intracellularly using CLSM and 3D-SIM. Our observations presented here, are representative images of a minimum of 20 biological replicate experiments performed on different days over the course of several years.

### Intracellular *M. hyopneumoniae* cells reside within vesicle-like structures

**Figure 2:**
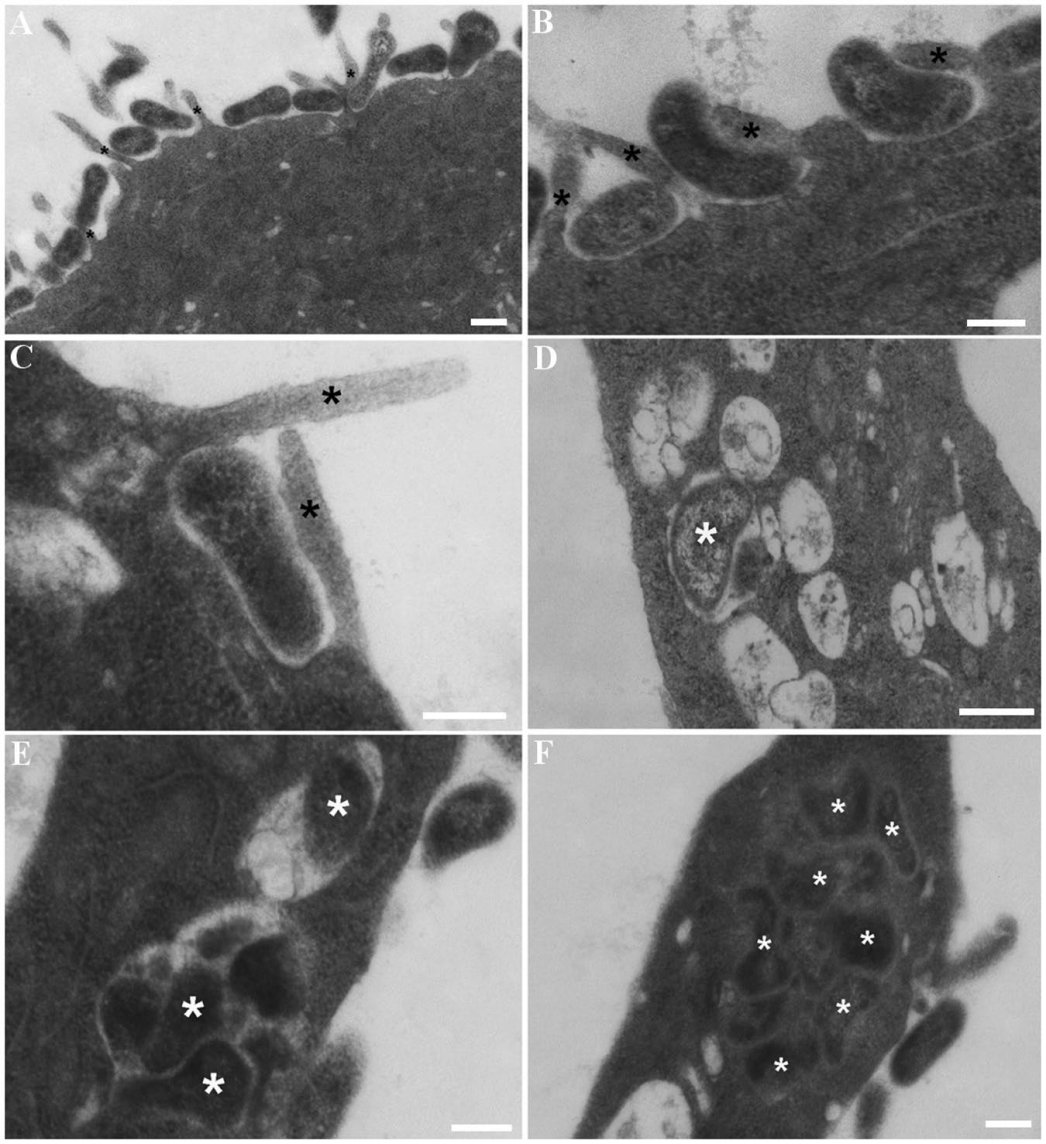
TEM of *M. hyopneumoniae* cells interacting with PK-15 cells. (**A–C**) Depict *M. hyopneumoniae* cells adhering to the surface of the PK-15 monolayer and being ushered across the membrane. Black asterisks (panels A, B and C) represent cell surface projections engulfing *M. hyopneumoniae* cells destined for uptake into the PK-15 cell. In panels D and E, white asterisks mark *M. hyopneumoniae* cells that have become internalised within electron-lucent vesicle-like structures. Some vesicles appear to contain membrane remnants of lysed *M. hyopneumoniae* cells. (**F**) Depicts *M. hyopneumoniae* cells (white asterisks) residing within the cytoplasm of PK-15 cells. The *M. hyopneumoniae* cells do not appear to be contained within a vesicle. Scale bars are 200 nm (**A**–**E**) and 400 nm (**F**).

Transmission electron microscopy (TEM) images also depict PK-15 cell surface projections engulfing *M*. *hyopneumoniae* cells (Fig. 2A–C). Once inside the PK-15 cells, *M. hyopneumoniae* cells were observed residing in electron-lucent, vesicle-like structures (Fig. 2D,E), indicating that the *M. hyopneumoniae* cells had become compartmentalised into endocytic vesicles. Additionally, they were also found free within the cytoplasm (Fig. 2F). Notably, Supplementary Fig. S3 depicts mycoplasma-like organisms residing within the cytoplasm of ciliated epithelium from the trachea of a pig infected experimentally with *M. hyopneumoniae*^32,38^. The electron-dense cells appear to be intact and do not seem to be associated with any vesicle-like structures (Supplementary Fig. S3). To our knowledge, this is the first experimental evidence depicting mycoplasma-like organisms residing within swine respiratory epithelial cells.

### *M. hyopneumoniae* cells are engulfed by clathrin- and caveolae-mediated endocytosis and are trafficked intracellularly via the complete endocytic pathway

**Figure 3:**
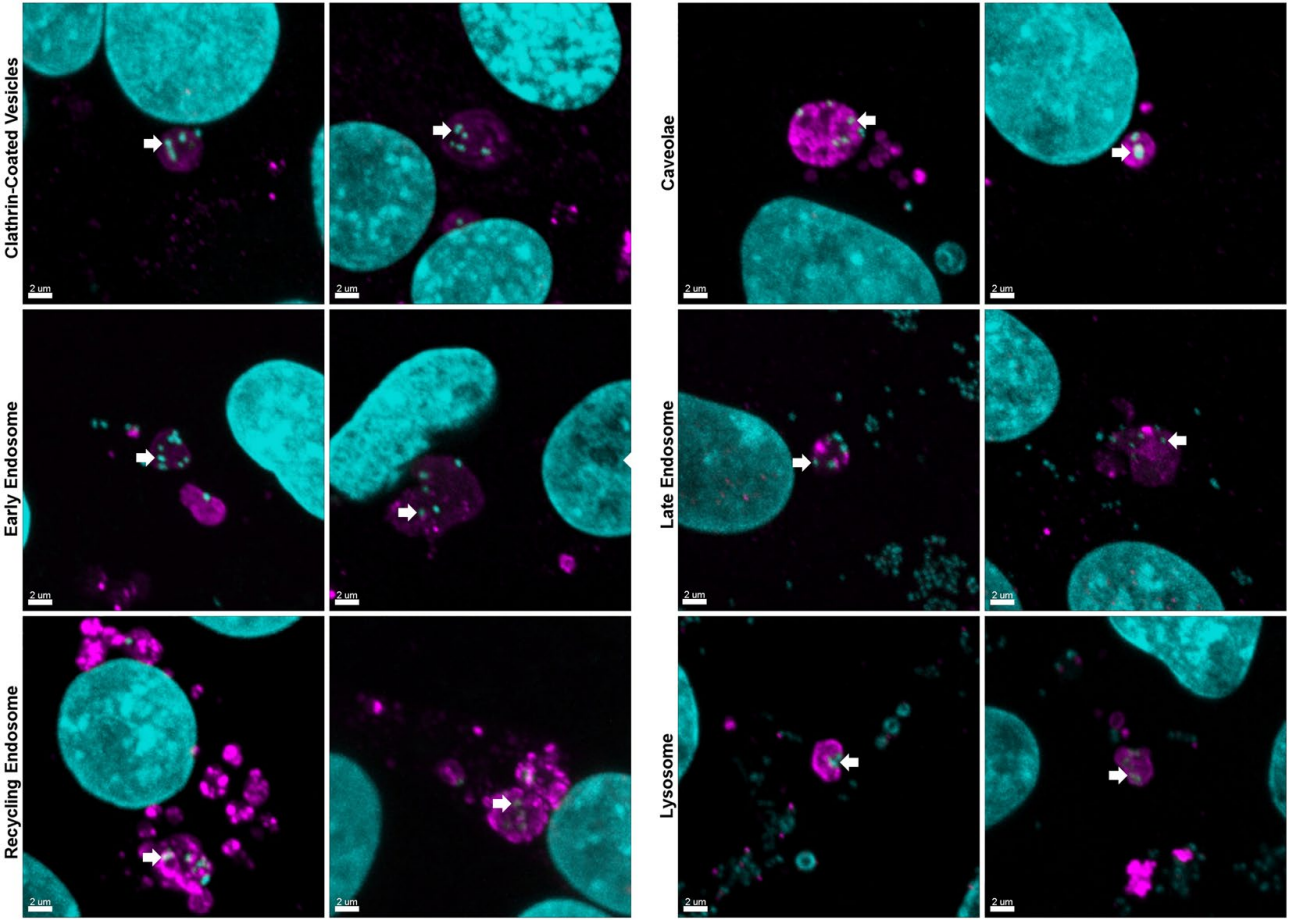
*M. hyopneumoniae* is trafficked intracellularly via the endocytic pathway. Confocal microscopy was used to image PK-15 monolayers infected with *M. hyopneumoniae*. Prior to permeabilisation, *M. hyopneumoniae* cells adhering to the PK-15 cell surface were labelled using F2_P94-J_ antisera conjugated to CF 488 (not shown in the fields of view). After permeabilisation, clathrin-coated vesicles (CCVs) (mAb_clath._), caveosomes (mAb_cav._), early (RAB5, left image and EEA1, right image), late (RAB7) and recycling (RAB11) endosomes, and lysosomes (LAMP1) were labelled and conjugated to CF 568 (magenta). Intracellular *M. hyopneumoniae* cells and PK-15 nuclei were stained using DAPI (cyan). *M. hyopneumoniae* cells are identified during all stages of the endocytic pathway (white arrows).

Clathrin- and caveolae- mediated endocytosis represent two endocytic pathways involved in the uptake of bacteria by eukaryotes^39^. We used confocal microscopy with monoclonal antibodies that recognise clathrin (mAb_clath._) and caveolin-1 (using mAb_cav_) to show *M. hyopneumoniae* cells residing within clathrin-coated vesicles (CCVs) and caveosomes (Fig. 3), respectively, after 16 h incubation with PK-15 monolayers. Notably, we observed CCVs in direct contact with the PK-15 membrane in close proximity to *M. hyopneumoniae* cells on the extracellular side of the membrane in the process of being engulfed (Supplementary Fig. S4). Furthermore, structures resembling caveolae in areas where *M. hyopneumoniae* cells were adhering to the PK-15 cell surface were observed via SEM (Supplementary Fig. S5).

To investigate the association of *M. hyopneumoniae* cells with the endocytic pathway, we stained infected PK-15 cells with a panel of antibodies specific for early, late and recycling endosomes, and lysosomes. *M. hyopneumoniae* cells residing within early endosomes that display the markers EEA1 and RAB5 were observed using confocal microscopy (Fig. 3). *M. hyopneumoniae* cells were also observed within endosomes displaying RAB11 suggesting that *M. hyopneumoniae* can influence the endosomal pathway and be exported back to the extracellular milieu (Fig. 3). Early endosomes mature into late endosomes displaying RAB7, and *M. hyopneumoniae* cells were also observed within these vesicles (Fig. 3). Typically, late endosomes fuse with lysosomes to allow the delivery of hydrolytic enzymes to degrade the engulfed cargo. Using LAMP1 as a marker of the lysosomal membrane, we detected numerous lysosomes within the cytoplasm of PK-15 cells containing intracellular *M. hyopneumoniae* cells (Fig. 3). In these examples, numerous extracellularly adhering *M. hyopneumoniae* cells could be seen in the vicinity of the vesicle-bound intracellular bacteria, further supporting our differential staining protocol (Supplementary Fig. S6). Notably, *M. hyopneumoniae* cells were identified using 3D-SIM in the immediate vicinity of LAMP1-labelled lysosomal membrane fragments (Supplementary Fig. S7) and *M. hyopneumoniae* cells were identified in the cytoplasm of PK-15 cells (Fig. 2F–H) using TEM. To our knowledge, this is the first depiction of *Mycoplasma* spp. being trafficked intracellularly via the complete endocytic pathway.

### Fibronectin and integrin β1 are targets for *M. hyopneumoniae*

Previously we determined that the expression of host cell fibronectin is induced in PK-15 cells at the place where *M. hyopneumoniae* makes contact with the membrane and in the ciliated epithelium lining of the upper respiratory tract of swine following experimental infection with *M. hyopneumoniae*^14^. In uninfected PK-15 cells, fibronectin radiates with a fibril-like structure ahead of the advancing edge of PK-15 cells by as much as 40 µm, localising at intercellular junctions between adjoining PK-15 cells, beneath the PK-15 monolayer (Supplementary Fig. S8). The pattern made by the radiating plumes of fibronectin were reminiscent of the pattern of localisation of *M. hyopneumoniae* during infection of PK-15 cells. In addition, regions ahead of the leading edge of PK-15 cells were locations where *M. hyopneumoniae* appeared to adhere to the glass surface (Supplementary Fig. S8). Other than regions in close proximity to the advancing edge of the PK-15 monolayer, *M. hyopneumoniae* does not appear to bind to glass surfaces. SEM of PK-15 cells infected with *M. hyopneumoniae* show the bacteria contacting material secreted onto the glass ahead of the leading edge, often along the length of fibres that are consistent with fibronectin (Supplementary Fig. S8). This was confirmed using confocal microscopy which showed *M. hyopneumoniae* cells adhering to fibronectin plumes secreted by PK-15 cells at the leading edge (Supplementary Fig. S8).

**Figure 4:**
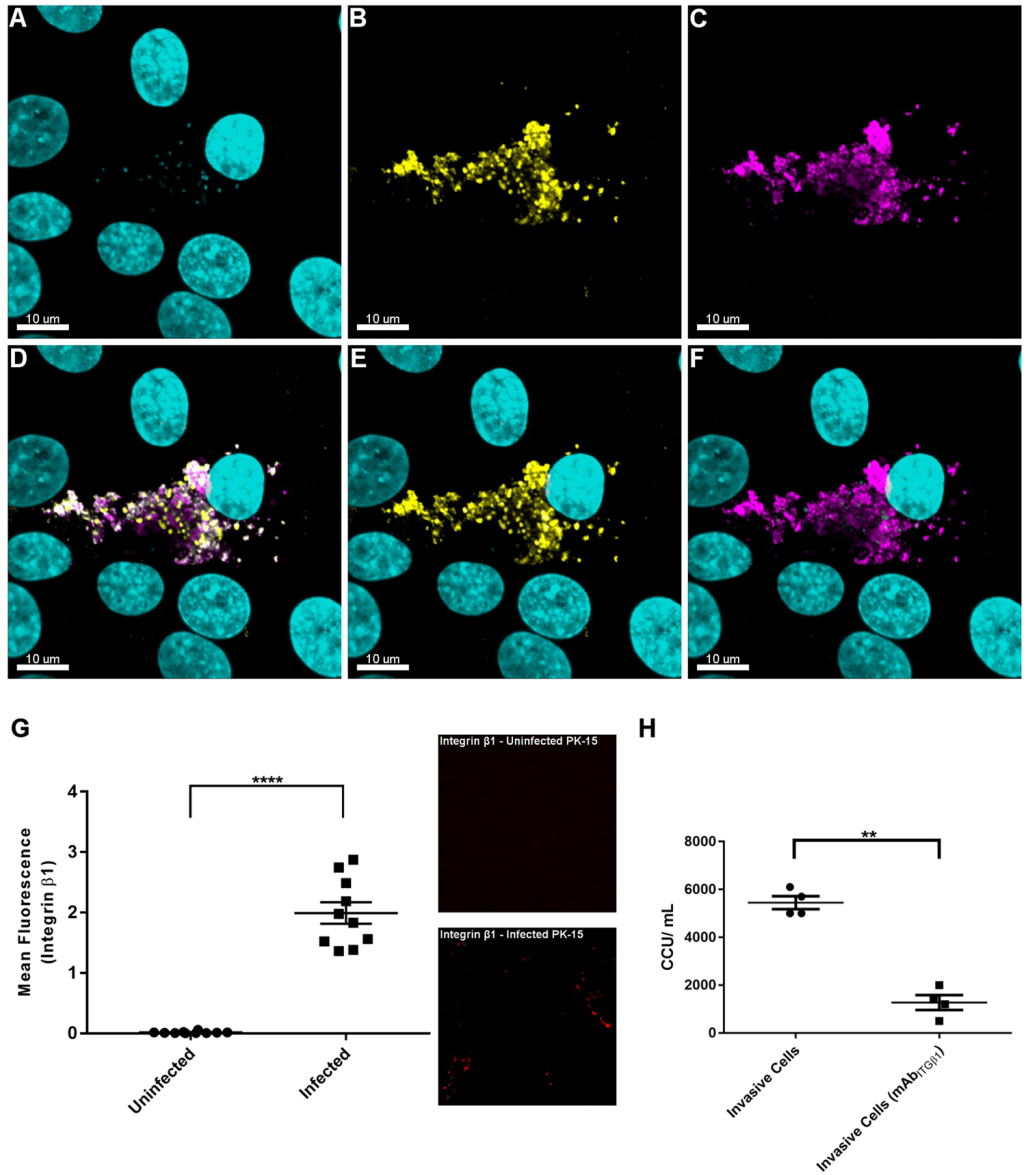
*M. hyopneumoniae* interacts with integrin β1 via fibronectin. (**A**–**F**) Confocal images of *M. hyopneumoniae*-infected monolayers. *M. hyopneumoniae* cells were stained DAPI (cyan), fibronectin was labelled with pAb_Fn_ conjugated to CF 488 (yellow) and integrin β1 was labelled with mAb_ITGβ1_ conjugated to CF 568 (magenta). Panels A–C show individual channels depicting *M. hyopneumoniae* cells (panel A), fibronectin expression (panel B) and expression of integrin β1 (panel C). The pattern of colonisation of *M. hyopneumoniae* cells on the surface of the PK-15 (panel A) mirrors the pattern localisation of fibronectin (panel B) and integrin β1 (panel C). Punctate fibronectin staining can be seen surrounding the *M. hyopneumoniae* cells (panels A,B,E) consistent with the results of an earlier study^14^. Integrin β1 forms pockets around the fibronectin-coated *M. hyopneumoniae* cells (panels C,D,F). (**G**) Depicts the mean fluorescence intensity corresponding to integrin β1 staining (10 random fields of view) of *M. hyopneumoniae*-infected PK-15 cells compared to uninfected controls. A representative image of each field of view is shown. Integrin β1 staining was significantly higher (p < 0.0001, indicated as****) in *M. hyopneumoniae*-infected PK-15 cells than in uninfected controls. The data is represented as individual data points with the standard error of the mean. (**H**) Depicts the results from our gentamicin protection assay and the effect of pretreating PK-15 cells with mAb_ITGβ1_ on the ability of *M. hypneumoniae* to invade PK-15 cells. The presence of mAb_ITGβ1_ prior to exposure of PK-15 cells with *M. hyopneumoniae* significantly reduces the number of *M. hyopneumoniae* cells recovered after gentamicin killing (an approximate 75% reduction). The data is presented as individual data points from four replicate experiments where each point represents the average of technical duplicates. The standard error of the mean is also shown. A paired t-test was performed with a *p* < 0.01, indicated as**.

In a previous study, *M. hyopneumoniae* cells were shown to sequester fibronectin to their cell surface^14^, a finding mirrored in this work (Fig. 4, panels B,E). In the extracellular matrix fibronectin connects with the actin cytoskeleton via the bridging molecule, integrin β1^40^. Numerous bacterial pathogens hijack these molecules to initiate internalisation^41^. Confocal microscopy was used to investigate co-localisation of fibronectin and integrin β1 in PK-15 cells infected with *M. hyopneumoniae* (Fig. 4A–F). Abundant fibronectin staining was observed to associate with regions on PK-15 cells that were colonised with *M. hyopneumoniae* (Fig. 4B,E), an observation that is consistent with these bacterial cells sequestering fibronectin onto their cell surface. In addition, it was noted that integrin β1 staining was abundant in the same areas, forming “pockets” around the fibronectin-bound *M. hyopneumoniae* cells (Fig. 4C–F). Consistent with this observation was significantly higher integrin β1 staining of *M. hyopneumoniae*-infected PK-15 cells compared to the uninfected control (Fig. 4G). Collectively, these data suggest that *M. hyopneumoniae* cells sequester fibronectin that is expressed early in response to infection and co-localises with integrin β1. To determine if integrin β1 plays a role in intracellular uptake of *M. hyopneumoniae*, PK-15 cells were pre-incubated with a neutralising integrin β1 antibody (mAb_ITGβ1_) for 2 h prior to infection with *M. hyopneumoniae*. Our modified gentamicin protection assay showed that the antibody blocked (~75% reduction in viable colonies) the ability of *M. hyopneumoniae* to become internalised within PK-15 cells (Fig. 4H). This is the first time that integrin β1 has been shown to play a role during the initial phase of cellular invasion by *M. hyopneumoniae*, or any other Mycoplasma species.

### *M. hyopneumoniae* induces cytoskeletal rearrangements in the porcine respiratory tract

**Figure 5:**
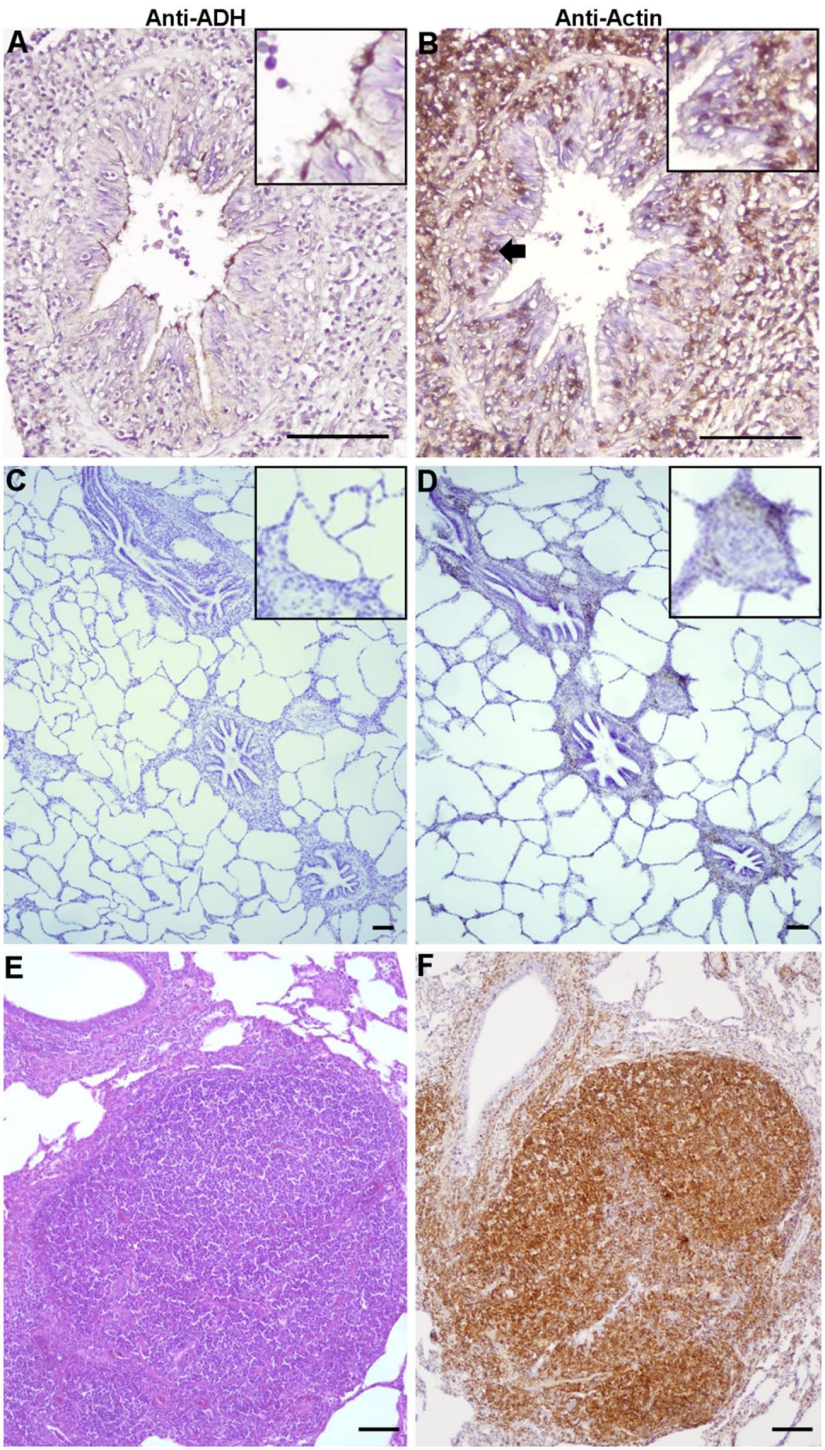
Immunohistochemistry of *M. hyopneumoniae*-infected, and uninfected, control tracheal tissue. Panels A and B depict serial sections of diseased tissue stained with anti-F2_P94-J_ (anti-ADH) and mAb_β-act_ (anti-actin), respectively. (**A**) *M. hyopneumoniae* cells labelled with anti-F2_P94-J_ localise at the ciliary border as described previously^14^. (**B**) Intense actin staining is observed in subepithelium tissue as well as the infiltration of intraepithelial leukocytes (black arrow) that stain with mAb_β-act_. (**C,D**) Depict healthy control tissue stained with anti-F2_P94-J_ and mAb_β-act_, respectively. In panel C, staining by anti-F2_P94-J_ is absent as expected and minimal staining is evident with mAb_β-act_ in panel D. As expected, there is no evidence of infiltration of intraepithelial leukocytes in the healthy control tissue. A lymphoid follicle from *M. hyopneumoniae*-infected tissue stained with haemotoxylin and eosin (panel E) and _mAbβ-act_ (panel F) shows the presence of intraepithelial leukocytes and intense actin staining.

The interaction of fibronectin with integrin β1 during the early stages of bacterial infection is known to induce cytoskeletal rearrangements in eukaryote cells that promote pathogen uptake^42^. However, it is not known if *M. hyopneumoniae* infection influences the expression of a key cytoskeletal protein, actin, in the porcine respiratory tract. To examine this, we stained serial tracheal sections with mAb_β-act_ and with F2_P94-J_ antisera. It was observed that serial tracheal sections from pigs infected experimentally with *M. hyopneumoniae* (diseased tissue) that were stained with F2_P94-J_ antisera contained bacterial cells adhering along the ciliary border of the epithelium (Fig. 5A) as expected. A serial section of the same diseased tissue stained with mAb_β-act_ identified actin quite prominently in the subepithelial layer (Fig. 5B), whereas control uninfected tissues did not stain intensely with mAb_β-act_ (Fig. 5C,D). In diseased tissue, lymphoid follicles and intraepithelial leukocytes that had infiltrated the tissue at the site of infection also stained intensely with mAb_β-act_ (Fig. 5E,F). These data suggest that extensive cytoskeletal rearrangements may occur in the respiratory tract of swine infected with *M. hyopneumoniae*.

### *M. hyopneumoniae* is trafficked alongside fibronectin and integrin β1

**Figure 6:**
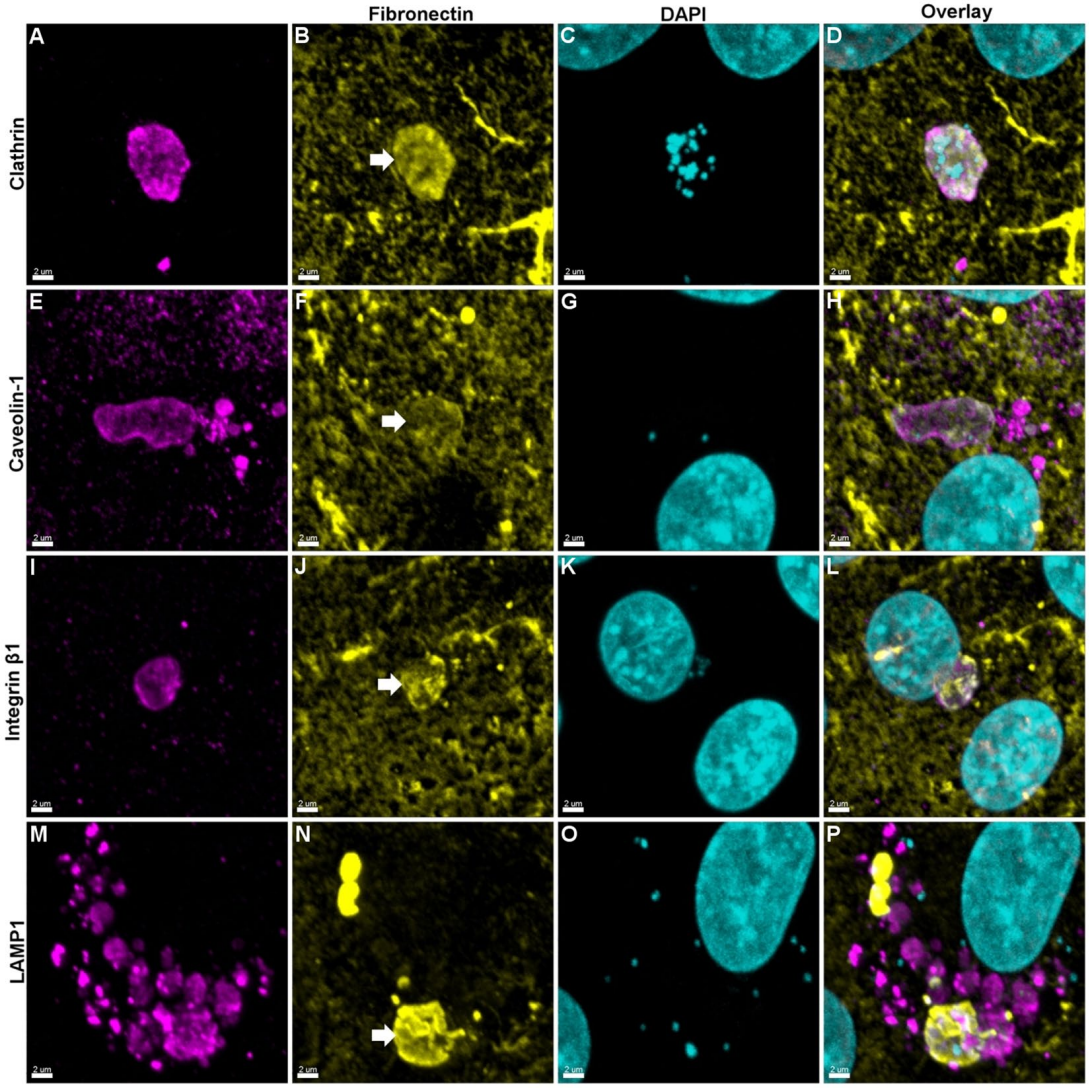
*M. hyopneumoniae* cells are internalised and trafficked with fibronectin. *M. hyopneumoniae* cells were allowed to adhere to PK-15 cells for 16 h after which they were fixed, permeabilised and incubated with pAb_Fn_ which was conjugated to CF 488 (yellow). Samples were then incubated with mAbs targeting CCVs (mAb_clath._), caveosomes (mAb_cav._), integrin β1 (mAb _ITGβ1_) and lysosomes (LAMP1) and conjugated to CF 568 (magenta). PK-15 nuclei and *M. hyopneumoniae* nucleic acids were stained with DAPI (cyan). Each channel is presented as individual columns in addition to an overlay. Vesicles containing fibronectin are indicated by a white arrow and can be seen colocalising with CCVs, caveosomes, integrin β1, and lysosomes. *M. hyopneumoniae* cells can also be seen within each of these vesicles. These images indicate that *M. hyopneumoniae* cells are engulfed alongside fibronectin and integrin β1 by CCVs and caveosomes that eventually fuse with lysosomes. Fibronectin as part of the extracellular matrix can also be seen as yellow fibrous material.

Clathrin- and caveolin-mediated endocytosis is also how the integrin heterodimer, α5β1, the primary receptor of fibronectin, is endocytosed^43,44^. Our hypothesis is that *M. hyopneumoniae* cells are endocytosed while bound to fibronectin, therefore we determined the spatial relationship between intracellular *M. hyopneumoniae* cells coated with fibronectin and integrin β1, caveosomes, CCVs, and lysosomes. To visualise intracellular fibronectin, we incubated *M. hyopneumoniae*-infected (16 h) PK-15 cells with anti-fibronectin (pAb_Fn_) after membrane permeabilisation. It was found that *M. hyopneumoniae* cells associated both with CCVs (Fig. 6A–D) and with caveosomes (Fig. 6E–H) containing fibronectin. In one example, upwards of 20 *M. hyopneumoniae* cells were seen within a single CCV (Fig. 6C). Vesicle-like structures harbouring *M. hyopneumoniae* cells were also found to simultaneously stain with mAb_ITGβ1_ and fibronectin (Fig. 6I–L). Lysosomes containing fibronectin and *M. hyopneumoniae* cells were repeatedly observed (Fig. 6M–P). Numerous lysosomes were observed surrounding vesicle-like structures that contain both fibronectin and *M. hyopneumoniae* cells (Fig. 6). Secondary antibody controls ensured that the observed association of fibronectin and these vesicles was not due to antibody cross-reactivity (data not shown).

## Discussion

*M. hyopneumoniae* has historically been considered an extracellular pathogen despite numerous studies suggesting the contrary^30–32,45^. One study in particular, was able to recover *M. hyopneumoniae* from extrapulmonary sites during treatment with a therapeutic dose of marbofloxacin^30^. Notably, no extrapulmonary bacteria were recovered in the post-treatment period, despite re-isolation from the trachea of all pigs post-treatment and no evidence of environmental contamination^30^; indicative of *M. hyopneumoniae* reinfection. In further support of this hypothesis, numerous studies have demonstrated that *M. hyopneumoniae* has the ability to sequester plasminogen to its cell surface and facilitate its activation to plasmin; a potent serine protease that can degrade a range of ECM and cellular junction components^9,16–18,46–48^. Additionally, elevated levels of plasmin have been detected in bronchialviolar lavage fluid of pigs infected with *M. hyopneumoniae*^18,49^. The ability to utilise host plasmin is a hallmark of pathogens that have developed sophisticated mechanisms to invade host cells and to disseminate to distal tissue sites^50–54^.

For the first time we show *M. hyopneumoniae* has the capacity to enter host-derived epithelial cells (PK-15) via clathrin- and caveolae-mediated endocytosis. Membrane bound *M. hyopneumoniae* captured via these mechanisms are transported intracellularly via early, recycling and late endosomes (Fig. 3). Notably, a sub-population of cells appear to survive fusion with lysosomes and escape into the cytosol (Supplementary Fig. S7). However, it is not known how this invasive subpopulation of *M. hyopneumoniae* cells differs from the majority of adherent, non-invasive cells. Confocal microscopy studies shown here, the isolation of *M. hyopneumoniae* from infected PK-15 cells on Friis agar after treatment with a dose of gentamicin sufficient to kill extracellular populations of this pathogen, and 3D-SIM images depicting intracellular *M. hyopneumoniae* cells in the vicinity of lysosomal membrane remnants, all provide compelling evidence in support of a possible intracellular existence and lifestyle for this pathogen. Notably, intracellular membrane-bound *M. hyopneumoniae* were also observed to be trafficked via recycling endosomes, where they can re-enter the extracellular milieu. While we have attempted to identify *M. hyopneumoniae* residing within ciliated epithelium in the respiratory tract of pigs, the evidence presented is limited but sufficient to encourage further *in vivo* studies.

Integrins are highly abundant membrane bound receptors that link extracellular ligands, particularly from the extracellular matrix, to cytoskeletal actin in the cell. Endocytic trafficking has an important role in regulating the presentation of integrin receptors on the cell surface and has a direct impact on important intracellular signalling events^55^. Therefore, it is perhaps unsurprising that a central theme in bacterial pathogenesis involves the interaction between fibronectin-binding proteins on the surface of invasive bacterial pathogens, fibronectin and integrin α5β1 in a manner that influences integrin clustering, to elicit an intracellular signalling cascade^41,56^. *M. hyopneumoniae* cells display numerous fibronectin-binding adhesins on their surface^9,11,14,18^, an observation consistent with the co-localisation of fibronectin with *M. hyopneumoniae* during colonisation of PK-15 cells, as well as along the ciliated epithelial cell border of the lungs of pigs experimentally-infected with *M. hyopneumoniae*^14^. Here we extend these earlier observations and show that integrin β1 staining co-localises with the deposition of fibronectin, where *M. hyopneumoniae* cells adhere on the surface of PK-15 cells. We observed increased integrin β1 staining in PK-15 cells that were infected with *M. hyopneumoniae* as well as the subsequent co-localisation of fibronectin-coated extracellular *M. hyopneumoniae* cells to these areas (Fig. 4). Interestingly, unpublished data from our lab suggests that integrin β1 is not differentially expressed in infected monolayers, suggesting that the increased staining seen here may in fact be due to conformational changes in integrin β1 that enhance binding of mAb_ITGβ1_ after fixation^57^. This would possibly imply that these conformational changes are due to the presence of *M. hyopneumoniae* and its ability to sequester fibronectin, as it is a well-known phenomenon that pathogens utilise ECM proteins to interact with and activate integrins^58^. Previously, it has been shown that mAb_ITGβ1_ can activate integrin β1^59^, therefore this may explain why it was able to block invasion when incubated with live cells prior to infection with *M. hyopneumoniae*. Indeed, the ability of anti-integrin β1 antibodies to block cell invasion demonstrate the potentially significant role of integrin β1 in promoting the ability of *M. hyopneumoniae* to enter PK-15 cells. These observations led us to hypothesize that the uptake of *M. hyopneumoniae* into CCVs and caveosomes, and the subsequent fusion with lysosomes, is regulated by interactions between fibronectin recruited to the bacterial cell surface and integrin β1. Consistent with this view, we routinely observed *M. hyopneumoniae* cells residing within CCVs, caveosomes and lysosomes, in areas where fibronectin expression has been induced (Fig. 6). In some instances, we observed *M. hyopneumoniae* cells together with fibronectin residing within integrin β1-staining vesicles (Fig. 6I–L), suggesting that fibronectin-integrin complexes are trafficked with *M. hyopneumoniae* via the endosomal pathway. Integrin β1-fibronectin complexes are known to be readily engulfed via both clathrin- and caveolae-mediated endocytosis prior to degradation by lysosomes or recycling back onto the cell surface^43,55,60^. Although we did not observe *M. hyopneumoniae* co-localising with fibronectin within recycling endosomes, we do provide evidence that *M. hyopneumoniae* cells are exocytosed (Fig. 3).

*M. hyopneumoniae* expresses surface-accessible actin-binding proteins and may target extracellular actin as a receptor on the surface of epithelial cells^35^. As seen previously, sections of porcine lung tissue prepared from pigs infected with *M. hyopneumoniae* showed that the organism localised to the ciliated epithelial surface in the airway lumen (Fig. 5A)^14,18^. Similar sections stained with mAb_β-act_ antibodies reveal deposition of actin in the sub-epithelial tissues beneath and surrounding infection foci (Fig. 5B) at levels that are absent from control uninfected porcine lung tissue (Fig. 5C,D). Cytoskeletal rearrangements in epithelial and subepithelial cells^61–63^ are induced by proinflammatory cytokines TNF-α and IL-6, that are known to be up-regulated during infection caused by *M. hyopneumoniae*^32,64^. These same cytokines induce the recruitment of intraepithelial leukocytes via a process that requires cytoskeletal (actin) reorganisation. Leukocytes are an established portal for the dissemination of many pathogens to distal tissue sites^65–68^ and provide a protective niche from the immune system. *M. hyopneumoniae* promotes the infiltration of leukocytes that stain with mAb_β-act_ to foci of infection. The inflammatory response, a hallmark of lung infection caused by *M. hyopneumoniae*, may provide the organism with a mechanism to invade and survive professional phagocytic cells for dissemination to the liver, spleen, kidneys and lymph nodes^30–32^. However, further studies are needed to interrogate this hypothesis.

Collectively, our data suggests that *M. hyopneumoniae* potentially hijacks the intrinsic eukaryotic α5β1-fibronectin recycling pathway to gain access to host cells where it can persist intracellularly. This has important implications for the trafficking of *M. hyopneumoniae* from the respiratory tract to distal tissue sites and potentially *vice versa* after extended periods of dormancy. This study paves the way for the development of therapeutic strategies that seek to interfere or block the ability of this pathogen to persist in its only known host *Sus scrofa*.

## Materials and Methods

All data generated or analysed during this study are included in this published article (and its Supplementary Information files).

### Bacterial strains and culture

*Mycoplasma hyopneumoniae* strain 232 was grown in modified Friis medium as described previously^69^. Broth cultures were incubated at 37 °C for approximately 16 h until they reached mid-exponential phase and the medium turned orange. At this stage, cultures contain approximately 5 × 10^5^–1 × 10^6^ CFU/mL. For growth on solid agar, cells were plated onto Friis agar using a method described previously^70^.

### Gentamicin protection assay

Porcine kidney epithelial-like monolayers (PK-15) were grown to semi-confluency and split into 12-well microtitre plates (~10^5^ cells/well) and incubated overnight. For infection studies, a 16 h culture of *M. hyopneumoniae* strain 232 was pelleted, washed twice in PBS, and resuspended in 25 mM HEPES in DMEM containing 5% fetal bovine serum (infection medium) prior to incubation at 37 °C for 2 h. *M. hyopneumoniae* cells were added, so that each well received 0.5 mL representative volume (approximately 7.5 × 10^5^ CFU/mL) of the initial culture, and allowed to adhere to PK-15 cells for 16 h at 37 °C/5% CO_2_. Non-adherent cells were removed by washing in PBS, followed by incubation in 300 µg ml^−1^ gentamicin in DMEM (filter sterilised through pore size 0.2 µm) for 4 h at 37 °C/5% CO_2_. Duplicate samples (positive controls) were not incubated with gentamicin. The gentamicin was removed and cells were washed 5 × in PBS to remove any residual antibiotic. The cell dissociation solution TrypLE (Gibco, ThermoFisher Scientific) was added to each well and incubated at 37 °C for 20 minutes. Cells were then removed by gently pipetting the TrypLE 3–4 times. The cell suspension was serially diluted in Friis broth to 10^−1^ and 10^−2^. An aliquot (50 µl) of each dilution was pipetted onto 9 cm Friis agar plates, the liquid was allowed to air dry in a sterile environment for 10 minutes, and the plates were incubated at 37 °C/5% CO_2_ for at least 7 days. *M. hyopneumoniae* colonies were counted using a stereomicroscope (×40 objective). Inhibition experiments were performed by pre-incubating PK-15 cells with a 1:100 dilution of monoclonal antibodies against integrin β1 (mAb_ITGβ1._; ~5 µg ml^−1^ mAb, Abcam) prior to the addition of *M. hyopneumoniae* cells.

### Immunofluorescence microscopy of internalised *M. hyopneumoniae* cells

Experiments were performed as described previously^35^ with some minor modifications. Once samples were fixed and blocked, *M. hyopneumoniae* cells were incubated with polycloncal F2_P94-J_ rabbit antisera at a dilution of 1:500 for 1 h at RT. A 1:1000 dilution of anti-rabbit CF 488-labelled secondary antibody (Biotium) was incubated for 1 h at RT. Cells were permeabilised in 0.5% (v/v) Triton X-100 in PBS for 5 min. Samples were then re-incubated with F2_P94-J_ rabbit antisera at a dilution of 1:500 for 1 h at RT, followed by incubating with a 1:1000 dilution of anti-rabbit CF 568-labelled secondary antibody (Biotium) for 1 h at RT. DAPI (Roche) was then added for 5 minutes at RT to stain nucleic acids. Samples were then prepared as previously described^35^.

### Immunofluorescence of *M. hyopneumoniae* cells and clathrin, caveosomes, endosomes, integrin β1 and fibronectin

Experiments were performed identically to those described above except that intracellular *M. hyopneumoniae* cells were labelled exclusively with DAPI and not F2_P94-J_ antisera. Fibronectin was labelled prior to permeabilisation using polyclonal antisera raised in rabbit against fibronectin (pAb_Fn_) as described previously^14^. Mouse monoclonal antibodies mAbs (Abcam) against clathrin (ab2731, mAb_clath._1:75), caveolin-1 (ab17052, mAb_cav._; 1:75), integrin β1 (ab30388, mAb_ITGβ1_;1:50), RAB7 (ab50533, 1:300) or LAMP1 (ab25630, 1:20) were incubated post-permeabilisation overnight at 4 °C, and then incubated with a 1:1000 dilution of anti-mouse CF 488- or 568-conjugated secondary antibodies (Biotium) for 1 h at RT. Rabbit polyclonal antibodies (Abcam) against RAB5 (1:2000), EEA1 (1:1000) were incubated post-permeabilisation for 1 h at room temperature, and then incubated with a 1:1000 dilution of anti-mouse CF 488- or 568-conjugated secondary antibodies (Biotium) for 1 h at room temperature.

### Immunofluorescence microscopy of infected monolayers

Samples were imaged as previously described^35,71^, using a Nikon A1 Confocal Laser Scanning Microscope and a V3 DeltaVision OMX 3D-SIM Imaging System (Applied Precision, GE Healthcare).

### Image analysis

Images captured with the Nikon A1 Confocal microscope and those generated by the DeltaVision OMX 3D-SIM were processed using Bitplane, Imaris Scientific 3D/4D image processing software to create Maximum Intensity Projection (MIP) and slices images.

*M. hyopneumoniae*-infected PK-cells and uninfected controls were labelled with integrin β1 (described above) and 10 random fields of view were captured using an Olympus BX51 Upright Epi Fluorescence Microscope at 20 × magnification and a constant exposure. The mean fluorescence of integrin β1-stained samples was calculated, after thresholding and binary conversion, using ImageJ. GraphPad Prism 7 was used to plot the data, including the standard error of the mean, and to perform the statistical analyses (unpaired t-test).

### Infection of PK-15 cells for scanning electron microscopy

Experiments were performed as described previously with no modifications^35^.

*Preparation of tracheal sections and infected monolayers for transmission electron microscopy* Samples were fixed in a fixation solution containing 5% formaldehyde and 2% glutaraldehyde in cacodylate buffer (0.1 M cacodylate, 0.01 M CaCl2, 0.01 M MgCl2, 0.09 M sucrose, pH 6.9) and washed with cacodylate buffer. Samples were then osmificated with 1% aqueous osmium for 1 h at room temperature, washed and pellets of the samples were embedded in 2% water agar and cut into small cubes. Dehydration was achieved with a graded series of acetone (10%, 20%, 50%) for 30 min on ice followed by contrasting with 2% uranyl acetate in 70% acetone for overnight at 4 °C and further dehydrated with 90% and 100% acetone. Samples in 100% acetone were allowed to reach room temperature and were infiltrated with epoxy resin according to Spurr’s formular for a medium resin^72^; 1 part 100% acetone/1 part resin for overnight, 1 part 100% acetone/2 parts resin for 8 h, pure resin for overnight and several changes the following 2 days. Samples were then transferred to resin filled gelatine capsules and polymerized for 8 h at 75 °C. Ultrathin sections were cut with a diamond knife, picked up with formvar-coated copper grids (300 mesh) and counterstained with 2% aqueous uranyl acetate and lead citrate. After air-drying samples were examined in a Zeiss transmission electron microscope TEM910 at an acceleration voltage of 80 kV. Images were recorded digitally with a Slow-Scan CCD-Camera (ProScan, 1024 × 1024, Scheuring, Germany) with ITEM-Software (Olympus Soft Imaging Solutions, Münster, Germany). Brightness and contrast were adjusted with Adobe Photoshop CS4.

### Immunohistochemistry

All animal procedures were approved by the Animal Ethics Committee at the Elizabeth Macarthur Agricultural Institute and were in accordance with the Australian Code of Practice for the Care and Use of Animals for Scientific Purposes. Experiments were performed as previously described^14,32,38^ with a minor modification. Briefly, 19 male weaner pigs, sourced from a commercial herd, were infected endotracheally, with *M. hyopneumoniae* strain Hillcrest and euthanized 6 weeks post-infection. Serial sections of lung lesions from pigs, as well as healthy lung tissue from control pigs were examined for actin distribution by staining with 1:200 mAb_β-act_ for 1 hr at RT.

## Electronic supplementary material

Supplementary Figures

## References

[1] Chae C (2016). Porcine respiratory disease complex: Interaction of vaccination and porcine circovirus type 2, porcine reproductive and respiratory syndrome virus, and Mycoplasma hyopneumoniae. Veterinary journal.

[2] DeBey MC, Ross RF (1994). Ciliostasis and loss of cilia induced by *Mycoplasma hyopneumoniae* in porcine tracheal organ cultures. Infection and immunity.

[3] Holst S, Yeske P, Pieters M (2015). Elimination of *Mycoplasma hyopneumoniae* from breed-to-wean farms: A review of current protocols with emphasis on herd closure and medication. Journal of Swine Health and Production.

[4] Maes D (2008). Control of Mycoplasma hyopneumoniae infections in pigs. Veterinary microbiology.

[5] Mebus CA, Underdahl NR (1977). Scanning electron microscopy of trachea and bronchi from gnotobiotic pigs inoculated with *Mycoplasma hyopneumoniae*. American journal of veterinary research.

[6] Underdahl NR, Kennedy GA, Ramos AS (1980). Duration of *Mycoplasma hyopneumoniae* infection in gnotobiotic pigs. *The Canadian veterinary journal*. La revue veterinaire canadienne.

[7] Pendarvis K (2014). Proteogenomic mapping of Mycoplasma hyopneumoniae virulent strain 232. BMC genomics.

[8] Berry IJ (2017). N-terminomics identifies widespread endoproteolysis and novel methionine excision in a genome-reduced bacterial pathogen. Scientific reports.

[9] Bogema, D. R. *et al*. Characterization of cleavage events in the multifunctional cilium adhesin Mhp684 (P146) reveals a mechanism by which *Mycoplasma hyopneumoniae* regulates surface topography. *mBio* **3**, 10.1128/mBio.00282-11 (2012). 10.1128/mBio.00282-11 PMC3322551 22493032

[10] Bogema DR (2011). Sequence TTKF ↓ QE defines the site of proteolytic cleavage in Mhp683 protein, a novel glycosaminoglycan and cilium adhesin of *Mycoplasma hyopneumoniae*. The Journal of biological chemistry.

[11] Deutscher AT (2010). Repeat regions R1 and R2 in the P97 paralogue Mhp271 of *Mycoplasma hyopneumoniae* bind heparin, fibronectin and porcine cilia. Molecular microbiology.

[12] Deutscher AT (2012). *Mycoplasma hyopneumoniae* Surface proteins Mhp385 and Mhp384 bind host cilia and glycosaminoglycans and are endoproteolytically processed by proteases that recognize different cleavage motifs. Journal of proteome research.

[13] Djordjevic SP, Cordwell SJ, Djordjevic MA, Wilton J (2004). & Minion, F. C. Proteolytic Processing of the Mycoplasma hyopneumoniae Cilium Adhesin. Infection and immunity.

[14] Raymond BB (2015). Proteolytic processing of the cilium adhesin MHJ_0194 (P123J) in *Mycoplasma hyopneumoniae* generates a functionally diverse array of cleavage fragments that bind multiple host molecules. Cellular microbiology.

[15] Raymond BB (2013). P159 from *Mycoplasma hyopneumoniae* binds porcine cilia and heparin and is cleaved in a manner akin to ectodomain shedding. Journal of proteome research.

[16] Seymour LM (2010). A processed multidomain *Mycoplasma hyopneumoniae* adhesin binds fibronectin, plasminogen, and swine respiratory cilia. The Journal of biological chemistry.

[17] Seymour LM (2011). Mhp107 is a member of the multifunctional adhesin family of *Mycoplasma hyopneumoniae*. The Journal of biological chemistry.

[18] Seymour LM (2012). Mhp182 (P102) binds fibronectin and contributes to the recruitment of plasmin(ogen) to the *Mycoplasma hyopneumoniae* cell surface. Cellular microbiology.

[19] Tacchi JL (2016). Post-translational processing targets functionally diverse proteins in *Mycoplasma hyopneumoniae*. Open biology.

[20] Tacchi JL (2014). Cilium adhesin P216 (MHJ_0493) is a target of ectodomain shedding and aminopeptidase activity on the surface of *Mycoplasma hyopneumoniae*. Journal of proteome research.

[21] Wilton J (2009). Mhp493 (P216) is a proteolytically processed, cilium and heparin binding protein of *Mycoplasma hyopneumoniae*. Molecular microbiology.

[22] Wyrsch E (2015). Comparative genomic analysis of a multiple antimicrobial resistant enterotoxigenic E. coli O157 lineage from Australian pigs. BMC genomics.

[23] Zhu W, Zhang X, Yang J, Xu W, Xu M (2015). Simultaneous determination of multi-classes of veterinary drug residues in pork by ultra performance liquid chromatography coupled with quadrupole-time of flight mass spectrometry. Se Pu.

[24] Hawkey PM (2015). Multidrug-resistant Gram-negative bacteria: a product of globalization. J Hosp Infect.

[25] Reid, C. J. *et al*. Porcine commensal Escherichia coli: a reservoir for class 1 integrons associated with IS26. *Microb Genom* **3**, 10.1099/mgen.0.000143 (2017). 10.1099/mgen.0.000143 PMC5761274 29306352

[26] Burki S (2015). Invasion and persistence of Mycoplasma bovis in embryonic calf turbinate cells. Veterinary research.

[27] McGowin CL, Popov VL, Pyles RB (2009). Intracellular Mycoplasma genitalium infection of human vaginal and cervical epithelial cells elicits distinct patterns of inflammatory cytokine secretion and provides a possible survival niche against macrophage-mediated killing. BMC microbiology.

[28] Yavlovich A, Katzenell A, Tarshis M, Higazi AA, Rottem S (2004). Mycoplasma fermentans binds to and invades HeLa cells: involvement of plasminogen and urokinase. Infection and immunity.

[29] Yavlovich A, Tarshis M, Rottem S (2004). Internalization and intracellular survival of Mycoplasma pneumoniae by non-phagocytic cells. FEMS microbiology letters.

[30] Le Carrou J, Laurentie M, Kobisch M, Gautier-Bouchardon AV (2006). Persistence of *Mycoplasma hyopneumoniae* in experimentally infected pigs after marbofloxacin treatment and detection of mutations in the parC gene. Antimicrobial agents and chemotherapy.

[31] Marois C, Le Carrou J, Kobisch M, Gautier-Bouchardon AV (2007). Isolation of Mycoplasma hyopneumoniae from different sampling sites in experimentally infected and contact SPF piglets. Veterinary microbiology.

[32] Woolley LK (2012). Evaluation of clinical, histological and immunological changes and qPCR detection of *Mycoplasma hyopneumoniae* in tissues during the early stages of mycoplasmal pneumonia in pigs after experimental challenge with two field isolates. Veterinary microbiology.

[33] Buttenschon J (1997). Microbiology and pathology of fibrinous pericarditis in Danish slaughter pigs. Zentralbl Veterinarmed A.

[34] Burnett TA (2006). P159 is a proteolytically processed, surface adhesin of *Mycoplasma hyopneumoniae*: defined domains of P159 bind heparin and promote adherence to eukaryote cells. Molecular microbiology.

[35] Raymond BBA (2018). Extracellular Actin Is a Receptor for *Mycoplasma hyopneumoniae*. Frontiers in cellular and infection microbiology.

[36] Zielinski GC, Young T, Ross RF, Rosenbusch RF (1990). Adherence of Mycoplasma hyopneumoniae to cell monolayers. American journal of veterinary research.

[37] Hannan PC, O’Hanlon PJ, Rogers NH (1989). *In vitro* evaluation of various quinolone antibacterial agents against veterinary mycoplasmas and porcine respiratory bacterial pathogens. Research in veterinary science.

[38] Woolley LK (2014). Evaluation of recombinant *Mycoplasma hyopneumoniae* P97/P102 paralogs formulated with selected adjuvants as vaccines against mycoplasmal pneumonia in pigs. Vaccine.

[39] Rohde M, Muller E, Chhatwal GS, Talay SR (2003). Host cell caveolae act as an entry-port for group A streptococci. Cellular microbiology.

[40] Henderson B, Nair S, Pallas J, Williams MA (2010). Fibronectin: a multidomain host adhesin targeted by bacterial fibronectin-binding proteins. FEMS microbiology letters.

[41] Boehm M (2011). Major host factors involved in epithelial cell invasion of Campylobacter jejuni: role of fibronectin, integrinbeta1, FAK, Tiam-1, and DOCK180 in activating Rho GTPase Rac1. Frontiers in cellular and infection microbiology.

[42] Hauck CR, Borisova M, Muenzner P (2012). Exploitation of integrin function by pathogenic microbes. Curr Opin Cell Biol.

[43] Shi F, Sottile J (2008). Caveolin-1-dependent beta1 integrin endocytosis is a critical regulator of fibronectin turnover. J Cell Sci.

[44] Pellinen T (2008). Integrin trafficking regulated by Rab21 is necessary for cytokinesis. Dev Cell.

[45] Yagihashi T, Nunoya T, Mitui T, Tajima M (1984). Effect of Mycoplasma hyopneumoniae infection on the development of Haemophilus pleuropneumoniae pneumonia in pigs. *Nihon juigaku zasshi*. The Japanese journal of veterinary science.

[46] Jarocki, V. M. *et al*. MHJ_0461 is a multifunctional leucine aminopeptidase on the surface of *Mycoplasma hyopneumoniae*. *Open biology* **5**, 10.1098/rsob.140175 (2015). 10.1098/rsob.140175 PMC4313372 25589579

[47] Robinson MW (2013). MHJ_0125 is an M42 glutamyl aminopeptidase that moonlights as a multifunctional adhesin on the surface of *Mycoplasma hyopneumoniae*. Open biology.

[48] Widjaja M (2017). Elongation factor Tu is a multifunctional and processed moonlighting protein. Scientific reports.

[49] Woolley LK, Fell SA, Djordjevic SP, Eamens GJ, Jenkins C (2013). Plasmin activity in the porcine airways is enhanced during experimental infection with *Mycoplasma hyopneumoniae*, is positively correlated with proinflammatory cytokine levels and is ameliorated by vaccination. Veterinary microbiology.

[50] Bhattacharya S, Ploplis VA, Castellino FJ (2012). Bacterial plasminogen receptors utilize host plasminogen system for effective invasion and dissemination. Journal of biomedicine & biotechnology.

[51] Lahteenmaki K, Edelman S, Korhonen TK (2005). Bacterial metastasis: the host plasminogen system in bacterial invasion. Trends in microbiology.

[52] Raymond BB, Djordjevic S (2015). Exploitation of plasmin(ogen) by bacterial pathogens of veterinary significance. Veterinary microbiology.

[53] Sanderson-Smith ML, De Oliveira DM, Ranson M, McArthur JD (2012). Bacterial plasminogen receptors: mediators of a multifaceted relationship. Journal of biomedicine & biotechnology.

[54] Peetermans M, Vanassche T, Liesenborghs L, Lijnen RH, Verhamme P (2016). Bacterial pathogens activate plasminogen to breach tissue barriers and escape from innate immunity. Critical reviews in microbiology.

[55] De Franceschi N, Hamidi H, Alanko J, Sahgal P, Ivaska J (2015). Integrin traffic - the update. J Cell Sci.

[56] Josse J, Laurent F, Diot A (2017). Staphylococcal Adhesion and Host Cell Invasion: Fibronectin-Binding and Other Mechanisms. Frontiers in microbiology.

[57] Su Y (2016). Relating conformation to function in integrin alpha(5)beta(1). Proceedings of the National Academy of Sciences of the United States of America.

[58] Hauck CR, Ohlsen K (2006). Sticky connections: extracellular matrix protein recognition and integrin-mediated cellular invasion by *Staphylococcus aureus*. Current opinion in microbiology.

[59] Stupack DG, Shen C, Wilkins JA (1994). Control of lymphocyte integrin function: evidence for multiple contributing factors. Cell Immunol.

[60] Caswell PT, Vadrevu S, Norman JC (2009). Integrins: masters and slaves of endocytic transport. Nat Rev Mol Cell Biol.

[61] Du L (2012). Actin filament reorganization is a key step in lung inflammation induced by systemic inflammatory response syndrome. American journal of respiratory cell and molecular biology.

[62] Campos SB (2009). Cytokine-induced F-actin reorganization in endothelial cells involves RhoA activation. American journal of physiology. Renal physiology.

[63] Koukouritaki SB (1999). TNF-alpha induces actin cytoskeleton reorganization in glomerular epithelial cells involving tyrosine phosphorylation of paxillin and focal adhesion kinase. Mol Med.

[64] Choi C (2006). Expression of inflammatory cytokines in pigs experimentally infected with Mycoplasma hyopneumoniae. Journal of comparative pathology.

[65] Thwaites GE, Gant V (2011). Are bloodstream leukocytes Trojan Horses for the metastasis of Staphylococcus aureus?. Nature reviews. Microbiology.

[66] Russmann H, Ruckdeschel K, Heesemann J (1996). Translocation of Yersinia enterocolitica through an endothelial monolayer by polymorphonuclear leukocytes. Infection and immunity.

[67] Coombes JL (2013). Motile invaded neutrophils in the small intestine of Toxoplasma gondii-infected mice reveal a potential mechanism for parasite spread. Proceedings of the National Academy of Sciences of the United States of America.

[68] Drevets DA (1999). Dissemination of Listeria monocytogenes by infected phagocytes. Infection and immunity.

[69] Scarman AL, Chin JC, Eamens GJ, Delaney SF, Djordjevic SP (1997). Identification of novel species-specific antigens of *Mycoplasma hyopneumoniae* by preparative SDS-PAGE ELISA profiling. Microbiology.

[70] Kobisch M, Friis NF (1996). Swine mycoplasmoses. Rev Sci Tech.

[71] Strauss MP (2012). 3D-SIM super resolution microscopy reveals a bead-like arrangement for FtsZ and the division machinery: implications for triggering cytokinesis. PLoS biology.

[72] Spurr AR (1969). A low-viscosity epoxy resin embedding medium for electron microscopy. Journal of ultrastructure research.

