# Supplementary material for: Mycoplasma hyopneumoniae resides intracellularly within porcine epithelial cells
Source: Sci Rep. 2018 Dec 6;8:17697. doi: 10.1038/s41598-018-36054-3 (PMC6283846; doi:10.1038/s41598-018-36054-3)
Supplement: Supplementary file 1 — Supplementary Figures [file 41598_2018_36054_MOESM1_ESM.docx]

*Mycoplasma hyopneumoniae* resides intracellularly within porcine epithelial cells

Raymond BBA^1^, Turnbull L^1^, Cheryl Jenkins^2^, Madhkoor R^1^, Schleicher I^3^, Uphoff CC^4^, Whitchurch CB^1^, Rohde M^2^ and Djordjevic SP^1*^

* Corresponding author: Steven. P. Djordjevic


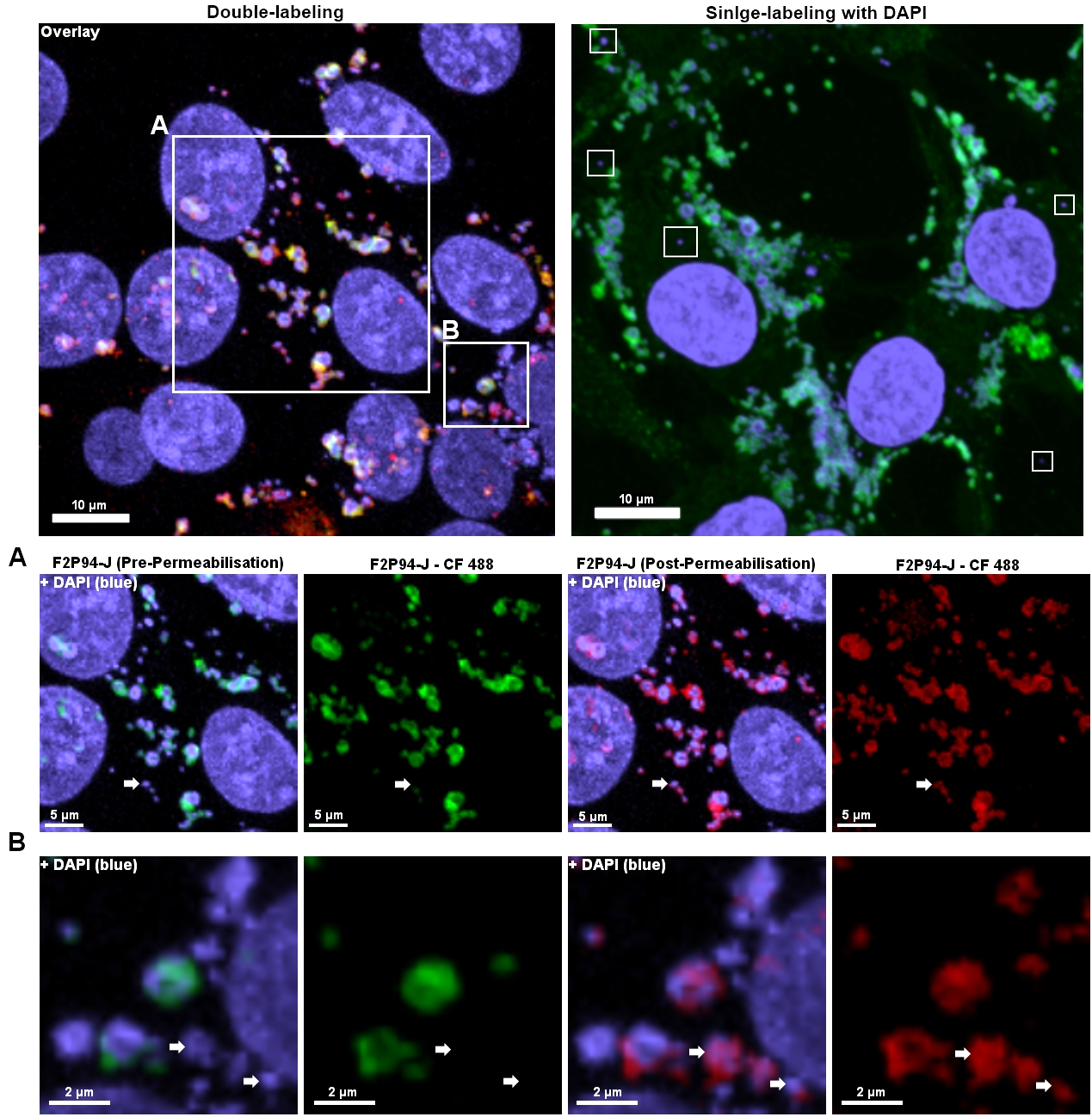


Supplementary Figure S1: Confocal micrographs of *M. hyopneumoniae* cells adhering to, and residing intracellularly within PK-15 monolayers after a 16 h incubation. Extracellular *M. hyopneumoniae* cells were labelled with rabbit F2_P94-J_ antisera followed by anti-rabbit CF 488, prior to permeabilisation. After permeabilisation, samples were re-incubated with F2_P94-J_ antisera, followed by incubating with CF 568. Using this technique, extracellular *M. hyopneumoniae* cells are double stained and appear yellow/green, and intracellular *M. hyopneumoniae* cells appear red. A comparative image using the single-labelling technique can be seen alongside the double-labelling technique. Extracellular *M. hyopneumoniae* cells were labelled with F2_P94-J_ antisera followed by anti-rabbit CF 488, prior to permeabilization and can be seen in green. Intracellular *M. hyopneumoniae* cells that were stained with DAPI post-permeabilisation can also be seen (white boxes).


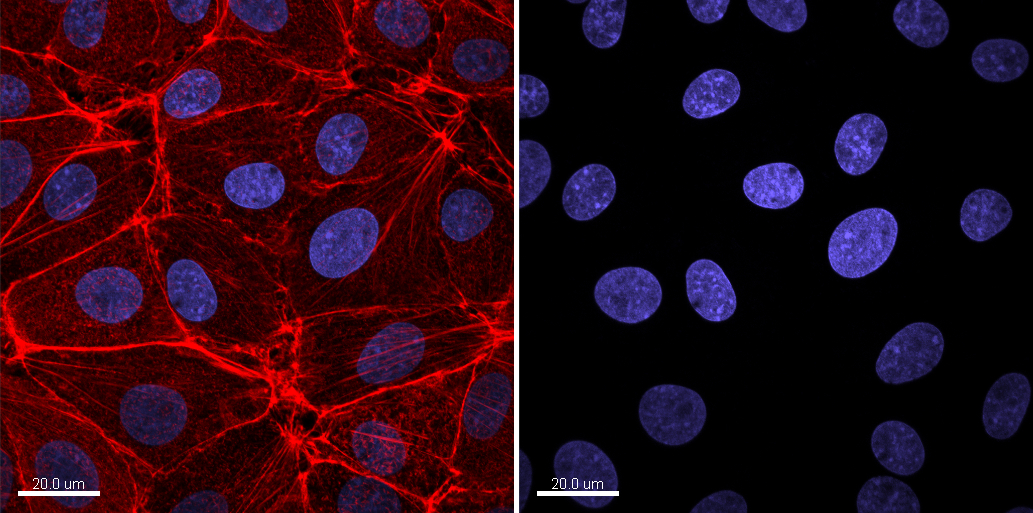


Supplementary Figure S2: Example of an uninfected control PK-15 monolayer. Cells were seeded onto coverslips and allowed to adhere and grow for 16 h prior. Samples were permeabilised and stained with DAPI (blue) to visualise nucleic acids and phalloidin (red) to visualise the cytoskeleton. Note that no DAPI-stained particles or bacteria can be observed.


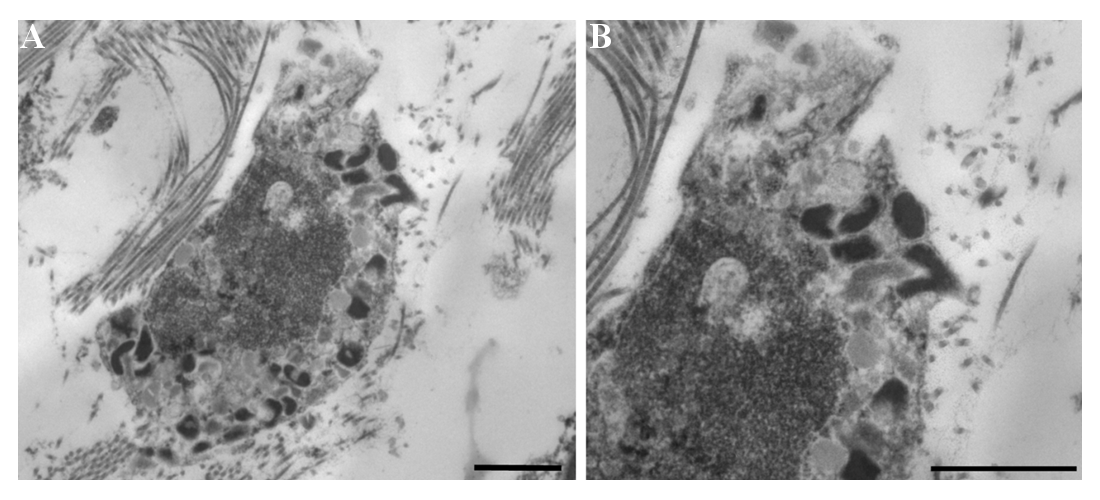


Supplementary Figure S3: Panels A and B show transmission electron micrographs of tracheal sections taken from the respiratory tract of a pig that was infected experimentally with *M. hyopneumoniae*. Arrows indicate *M. hyopneumoniae* cells which appear to reside intracellularly (black arrow). Cilia are also evident in these images. Panel B is a higher magnification of panel A. Scale bar is 2 µm.


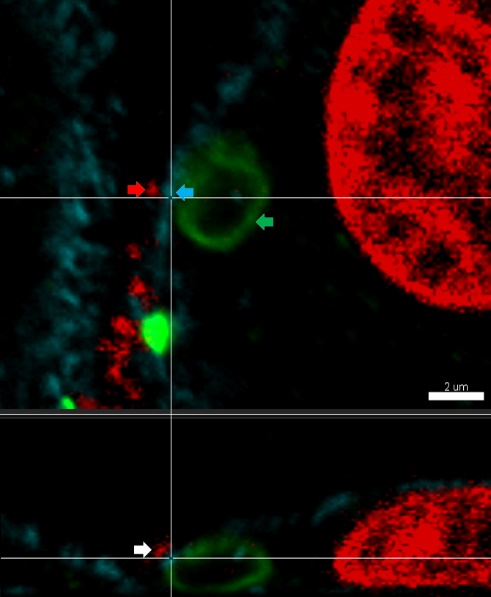


Supplementary Figure S4: Confocal micrograph of an *M. hyopneumoniae* cell being engulfed by a CCV. This orthogonal view shows numerous extracellular *M. hyopneumoniae* cells (red; stained with DAPI) closely associated with F-actin (blue; stained with phalloidin). This image also depicts a CCV (green arrow; labelled with mAb_clath._) associating closely with F-actin (blue arrow) near a single *M. hyopneumoniae* cell (red arrow). One possible interpretation is that the CCV has trafficked to the cell membrane beneath an *M. hyopneumoniae* cell that resides on the extracellular side of the membrane surface.


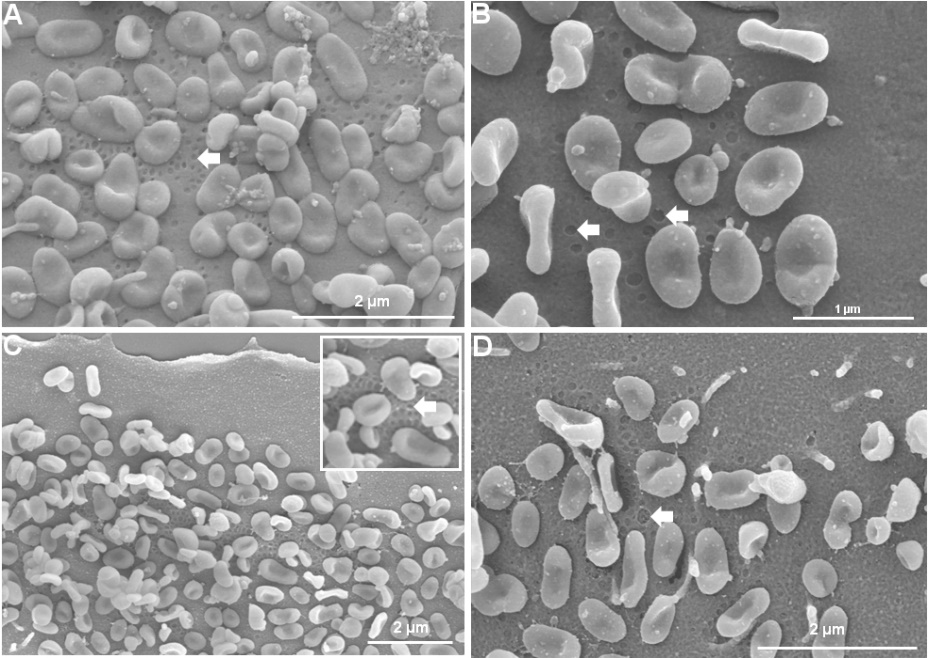


Supplementary Figure S5: *M. hyopneumoniae* cells associate with caveolae. A-D) SEM images of *M. hyopneumoniae* cells adhering to the PK-15 epithelial surface in close proximity to small invaginations (white arrows) in the PK-15 membrane that resemble caveolae. These structures range from 50 -100 nm.


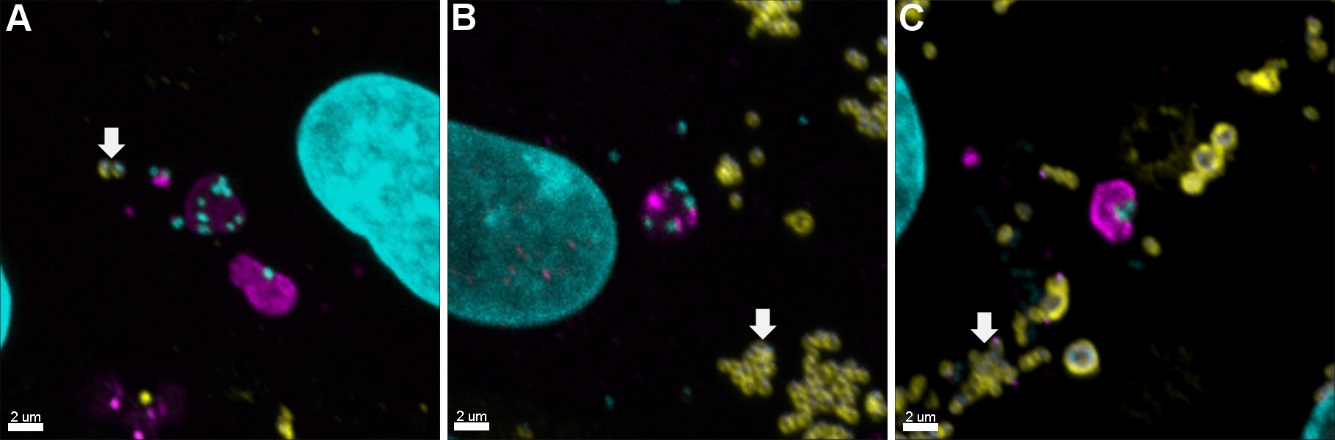


Supplementary Figure S6: Zoomed out images of RAB5, RAB7 and LAMP1 labelled samples from Fig. 3 demonstrating abundant, F2_P94-J_-labelled, extracellularly adhering bacteria (yellow).


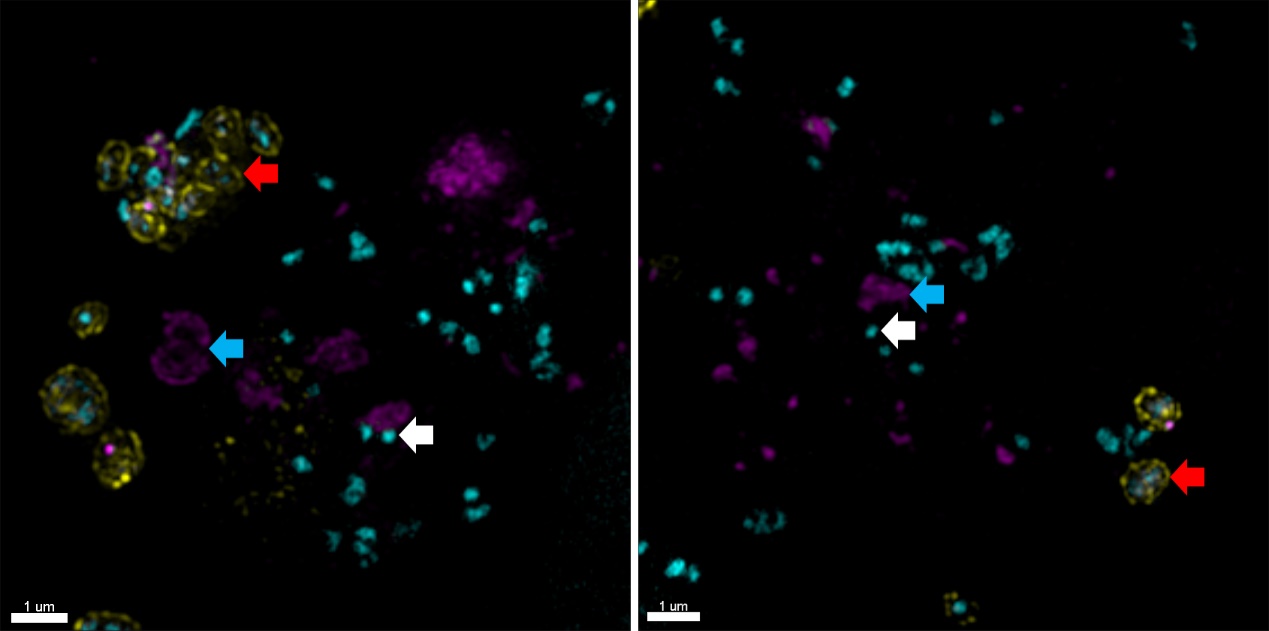


Supplementary Figure S7: 3D-SIM demonstrating *M. hyopneumoniae* cells residing within the cytosol of PK-15 cells. Extracellular *M. hyopneumoniae* cells, labelled with anti-F2_P94-J_ (yellow), can be seen adhering to the PK-15 cell (red arrows). Intracellular *M. hyopneumoniae* cells, stained with DAPI (cyan), can also be seen (white arrows). Remnants of destroyed lysosomes, labelled with anti-LAMP1 (magenta), are marked with blue arrows. The intracellular *M. hyopneumoniae* cells can be seen free in the cytoplasm in the proximity of lysosomal membrane remnants.


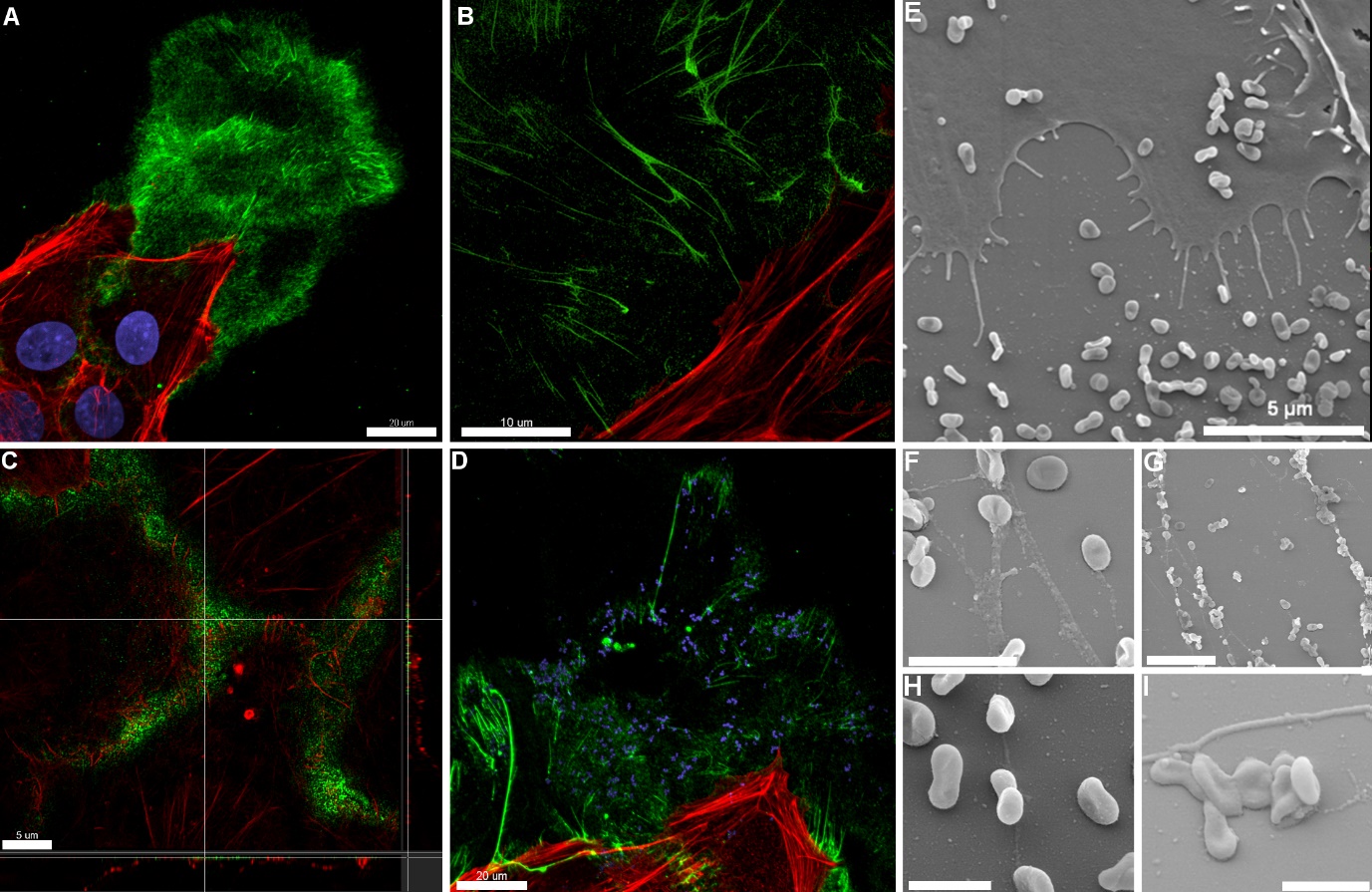


Supplementary Figure S8: IFM and SEM of *M. hyopneumoniae* adhering to secreted fibronectin. Panels A and B) Uninfected PK-15 monolayers were grown to semi-confluency, fixed, incubated with pAb_Fibronectin_, and conjugated to anti-rabbit CF™ 488 (green). Samples were permeabilised and stained with phalloidin (red) in panels A-D and DAPI (blue) in panels A and D to stain F-actin and nucleic acids respectively. Panel A demonstrates a confocal microscopy image of fibronectin (green) secreted ahead of the leading edge of the PK-15 cell monolayer. Panel B) 3D-SIM image showing that the “cloud-like” distribution of fibronectin is fibril-like. Panel C) 3D-SIM slice view showing fibronectin distributed at the intercellular junctions resides beneath the PK-15 monolayer. Panel D) Confocal image demonstrating *M. hyopneumoniae* cells (blue) adhering to fibronectin plumes secreted by a PK-15 cell. E-I) SEM images of *M. hyopneumoniae* cells adhering to fibronectin secreted onto the glass ahead of the leading edge of the PK-15 monolayer. *M. hyopneumoniae* cells can be seen in direct contact with this material, adhering along the length of the fibronectin fibres. Scale bars in F-I are 2 μm, 5 μm, 1 μm and 1 μm respectively.
